# The relationship between pregnancy count and duration of breast-feeding with metabolic syndrome (Fasa Persian cohort study)

**DOI:** 10.1186/s12905-023-02528-4

**Published:** 2023-07-13

**Authors:** Saeideh Zareei, Fatemeh Behrasi, Mohammad Mehdi Naghizadeh, Fatemeh Talebzadeh, Ali Kharmandar, Mojtaba Farjam, Reza Homayounfar

**Affiliations:** 1 Noncommunicable Diseases Research Center, Fasa University of Medical Sciences, Fasa, Iran; 2 Department Of Nutrition, School Of Medicine, Zahedan University Of Medical Sciences, Zahedan, Iran; 3 Student’s research committee, Fasa University of medical sciences, Fasa, Iran; 4 Faculty of Nutrition Sciences and Food Technology, National Nutrition and Food Technology Research Institute, Shahid Beheshti University of Medical Sciences, Tehran, Iran

**Keywords:** Metabolic syndrome, Pregnancy, Breast-feeding, Women, International diabetes Federation, Adult treatment panel III

## Abstract

**Background:**

Changes that occur during pregnancy and after that during breastfeeding induce some symptoms similar to metabolic syndrome (MetS) risk factors. This study aims to determine the relationship between pregnancy, as well as the duration of breastfeeding and MetS controlling the effect of other risk factors like hypertension, glucose intolerance, triglyceride, central obesity, and reduction of high-density lipoprotein in women of Fasa Persian Cohort Study.

**Materials and methods:**

In this cross-sectional study, 5015 women aged 35–70 years were investigated in the Sheshdeh region from 2016 to 2021, and the information related to the disease symptoms was collected through questionnaires, examinations, and laboratory tests. MetS was calculated based on two guidelines according to adult treatment panel III (ATP III) and international diabetes federation (IDF) methods. For reporting the data, the odds ratio with its 95% confidence interval was used. In order to eliminate the effect of confounders, logistic regression was used.

**Results:**

Prevalence of MetS showed a descending trend in women with up to two pregnancies and it reached 22.6% and 22.4% using ATPIII and IDF methods respectively, while with an increase in the number of pregnancies of more than two, MetS prevalence was ascending. The prevalence of MetS did not have any specific trend across various breastfeeding duration groups. Multivariate analysis approved that the odds ratio of developing MetS in comparison with women who had two pregnancies was significantly increasing trend when the pregnancy counts increased.

**Conclusion:**

The chance of developing MetS based on both IDF and ATP III methods after adjustment for confounding effects would grow with an increase in the number of pregnancies to more than two and breast-feeding of more than seven years. It is recommended that women with more than two pregnancies or the long duration of breast-feeding women undergo a specialized examination to investigate and control MetS problems so that future diseases could be prevented.

**Supplementary Information:**

The online version contains supplementary material available at 10.1186/s12905-023-02528-4.

## Background

Metabolic syndrome (MetS) refers to concurrent symptoms of hypertension, glucose intolerance, elevated blood triglyceride, central obesity, and reduction of blood high density lipoprotein (HDL). MetS is a major public health concern whose prevalence has been growing in both developed and developing countries, and is considered as a well-known risk factor for developing of type II diabetes, cardiovascular disease, and their resulting mortalities [1–3]. The prevalence of MetS has been reported 10–50% globally [4] and 32% in Iran [5]. The results obtained from most previous studies indicate greater prevalence of this disorder in women and the elderly. The factors affecting prevalence of MetS include insulin resistance, abdominal obesity, dyslipidemia, glucose intolerance, hypertension, pro-inflammatory state, genetic factors, intrauterine growth restriction, urban and sedentary lifestyle, diet, social, economic, and cultural factors, level of education, psychosocial stressors, living environment, and lifestyle [1, 4].

During pregnancy, changes occur including insulin resistance, atherogenic dyslipidemia, fat accumulation, and inflammatory process in order to ensure supply of energy and nutrients required for the fetus in the body of the pregnant mother, which are similar to MetS symptoms [6, 7]. With initiation of breast-feeding, metabolism of the mother’s body and insulin resistance improve. During pregnancy, a mechanism is established by which the control of prolactin - the lactation hormone - may regulate adipocyte biology, glucose and lipid metabolism, and protect women against type 2 diabetes after childbirth [8, 9]. It has also been found that in animal and human models, insulin-sensitive gene expression is dramatically up-regulated during the lactation cycle. Because insulin has a direct role in lactation, including essential roles in secretory differentiation, secretory activation, and mature milk production [10, 11]; Longer duration of breastfeeding may be associated with reduced risk of diabetes [12]. Also, the weight along with levels of triglyceride and blood glucose decrease, while HDL level also increases. However, weight gain or loss in the postpartum period has not yet been agreed upon. It seems that after breast-feeding cessation, the changes that occur for MetS symptom improvement continue for years [6, 13]. Researchers have concluded that there is a significant relationship between reduction of the rate of developing MetS and increase of duration of breast-feeding, indicating a dose-dependent correlation [14, 15]. During breastfeeding, the mother consumes is actually higher than 500 kcal (745+/-130 g/d) and it depends on the stage of breastfeeding and the infants’ growth and the deriving energy from their fat stores accumulated during pregnancy [15, 16].

Previous studies have shown a direct relationship between breastfeeding in reducing the prevalence of metabolic syndrome [17–20] and its components and the opposite result [21].

Pregnancy can lead to heart diseases in both direct and indirect ways. Being pregnant, along with factors such as obesity, blood pressure, high cholesterol, consumption and smoking, has been introduced as one of the factors of heart diseases [22, 23]. In addition to getting pregnant, the consequences of pregnancy such as early menstruation, early menopause, less in the first birth and a history of abortion, stillbirth or hysterectomy are independent factors of CVD [23].

Fetal survival is the result of some cardiometabolic changes during pregnancy, such as weight gain, dyslipidemia, increased plasma glucose, and insulin resistance, but all of the above directly increase the risk of CVD. Among other factors that increase the risk of cardiovascular disease during pregnancy, we can mention increased stress, endothelial dysfunction, inflammation, and the process of homeostasis [24–26].

Previous studies showed conflicting results regarding the relationship between parity and cardiometabolic health [27–31]. However, in women with a history of pregnancy and grand multiparity, the risk of CVD, MI, and type 2 diabetes increases, although prolonged breastfeeding may offset this risk [32].

The global growing mortality resulting from metabolic diseases including metabolic syndrome on the one hand as well as the preventability of this group of diseases with simple measures and interventions including establishment of screenings and modification of lifestyle have prompted increasing demands and attention for this group of diseases. Considering the importance of MetS regarding incidence of short-term and long-term consequences for the mother as well as its relationship with duration of pregnancy and breast-feeding, this research was done to determine the relationship between frequencies of pregnancy as well as breast-feeding duration and MetS among 35-70-year-old women in Fasa Persian Cohort Study.

## Materials and methods

The present cross-sectional research was performed on 35-70-year-old women referring to Fasa Town cohort center. The data collected from the first phase of Fasa cohort study were employed in this research. Fasa cohort is a branch of PERSIAN cohort (Prospective Epidemiological Research Studies in Iran) initiated from 2016, whose aim is to evaluate, identify noncommunicable disease risk factors, as well as calculate the risk of developing noncommunicable diseases in rural regions [33].

**Chart 1:**
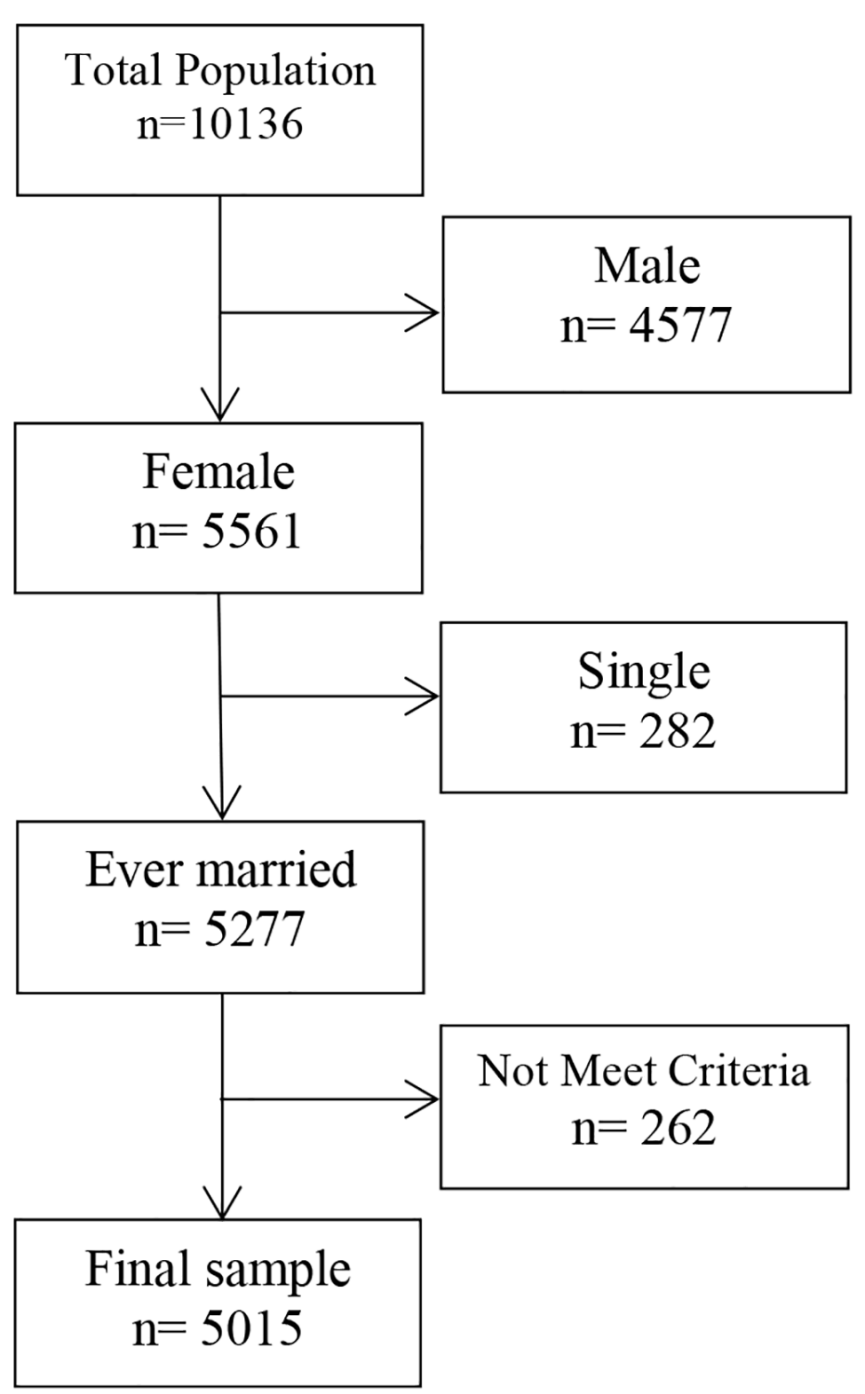
Diagram of participants in this study. The ever-married female participants of the Fasa Persian cohort Study who had complete data for MetS and met our study criteria were 5015 persons

The food frequency questionnaire (FFQ) was used to calculate the energy intake. The modified FFQ is administered in this phase to evaluate the eating habits and foods consumed by the participants. The FFQ is a semi-quantitative 125-item inventory. The inventory is used to obtain information on dietary intake over a 1-year period and is a Willett format questionnaire [34] modification based on Iranian food items. It includes a list of foods (with standard serving sizes) commonly consumed by Iranians. Individuals are requested to report their frequency of consumption of a given serving of each food item during the past year, on a daily, weekly, monthly or yearly basis. A standard portion size is designated for each item by using United States Department of Agriculture (USDA) serving sizes (e.g. bread, one slice; dairy, one cup).

The information in the Persian cohort was collected via questionnaire which included 18 topics. The titles of each section included: demographic characteristics, socioeconomic status, occupational background, status of place of residence, lifestyle, questions related to history of pregnancy (specific to women), history of chronic diseases, history related to drugs consumed, familial history of diseases, blood pressure measurement, international physical activity questionnaire (IPAQ), as well as frequency of food consumption and dietary habits [33]. The daily energy intake includes the percentage of received energy obtained from protein, carbohydrate, and fat, evaluated in each meal and snack via a one-year food reminder. The one-year food reminder records the food intakes reported by the person for one year on a meal-by-meal basis [33]. Each of them is completed in separate sections and by an experienced surveyor with mastery over the subject.

Socio-economic level questionnaire was completed to assess the wealth status for individuals and became a variable by principle component analysis (PCA) [33, 35, 91]. And classified according to the median during the analysis. ever smoker: An adult who has smoked at least 100 cigarettes in his or her lifetime, and who now smokes every day.

The extent of physical activity was obtained through the surveyors interviewing the participants via international physical activity questionnaire. This 24-hour questionnaire records the intensity of physical activity as metabolic equivalent (MET) during 24 h. In order to calculate the metabolic equivalent, the time spent for the activities mentioned in the questionnaire should be multiplied by the coefficient of that activity. The sum of numbers obtained from the hours allocated to physical activities by its MET coefficient leads to calculation of MET.h/day [36, 37].

In this Reashearch, MetS was determined by the researcher using available laboratory information and anthropometric indicators measured by the participants.

MetS was calculated according to the definition by the International Diabetes Federation (IDF) and separately based on the definition of hypercholesterolemia assessment and treatment among adults known as adult’s treatment panel (III) (ATP III). In IDF criterion, those who have obesity with two items or overall more than four items of the following elements are considered to have MetS. Also, based on ATP III definition, existence of three elements or more of these five diagnostic elements in a person is considered MetS. The diagnostic elements of MetS include fasting blood sugar (FBS) equal to an greater than 100 mg/dl or consumption of drugs for hyperglycemia, waist circumference (WC) of equal to or greater than 80 cm in women, triglyceride level (TG) equal to or greater than 150 mg/dl or taking drugs for high triglyceride, high density lipoprotein (HDL) level of equal to or lower than 50 mg/dl for women or taking drugs for low HDL levels, high systolic blood pressure (SBP) of equal to or greater than 130 and diastolic blood pressure (DBP) of equal to or greater than 85 mmHg, or taking drugs for controlling hypertension [1]. Furthermore, concerning the number of pregnancies or existence of current pregnancy and total years of breast-feeding, the participants’ information was collected through self-expression.

The collected data were analyzed by IBM SPSS 22 (IBM CO, Armonk, NY). For reporting the data, mean and standard deviation descriptive statistics were used along with odds ratio with 95% confidence interval.

In order to control the confounding effect, logistic regression was used. In this analysis, existence or absence of impairments in any of the MetS elements, as well as having or not having MetS were each chosen separately as the dependent variable. On the other hand, the set of variables of age, MET, energy, the representative index for socioeconomic variables, age of first marriage, and cigarette smoking were chosen as the independent variables. The history of the two pregnancies and 1–2 years of breastfeeding duration was chosen as the reference index [33]. Probability lower than 0.05 was chosen as the significance threshold. By adjusting these factors in the logistic regression model, their possible bias was resolved and the biased effect of these factors in incidence of MetS was prevented.

## Results

Out of about 12,000 volunteers referring to the cohort center, half of them were male and the other half were female. From 5509 women (54.3% of the total population participating in the Fasa Persian cohort study), 5015 had complete demographic information, and according to the inclusion criteria, they were eligible to be included. The mean age of these people was 48.63 ± 9.53 years with age range of 35–70 years. Based on the performed analyses, 42.3 and 42.7% of the studied individuals had MetS according to IDF and ATP III methods respectively. Other demographic variables are detailed in Tables 1 and 2.

**Table 1:** The frequency of MetS and demographic variables

Qualitative variable		MetS (NCEP ATP III criteria)		MetS (IDF criteria)		Total (n = 5015)
		No (n = 2876)	Yes (n = 2139)	No (n = 2894)	Yes (n = 2121)	
		Count(%)	Count(%)	Count(%)	Count(%)	
Marital status	Married	2551 (58.9%)	1781 (41.1%)	2566 (59.2%)	1766 (40.8%)	4332
	Widowed	265 (44.7%)	328 (55.3%)	268 (45.2%)	325 (54.8%)	593
	Divorced	60 (66.7%)	30 (33.3%)	60 (66.7%)	30 (33.3%)	90
Education	less than diploma	2726 (56.4%)	2105 (43.6%)	2744 (56.8%)	2087 (43.2%)	4831
	diploma	113 (82.5%)	24 (17.5%)	113 (82.5%)	24 (17.5%)	137
	higher than diploma	36 (78.3%)	10 (21.7%)	36 (78.3%)	10 (21.7%)	46
Occupation status	no	2175 (54.9%)	1789 (45.1%)	2189 (55.2%)	1775 (44.8%)	3964
	yes	701 (66.7%)	350 (33.3%)	705 (67.1%)	346 (32.9%)	1051
Ever Smoker	no	2757 (57.9%)	2002 (42.1%)	2774 (58.3%)	1985 (41.7%)	4759
	yes	119 (46.5%)	137 (53.5%)	120 (46.9%)	136 (53.1%)	256
**Quantitative variable**		**Mean** ± **SD**	**Mean** ± **SD**	**Mean** ± **SD**	**Mean** ± **SD**	**Total** **Mean** ± **SD**
Age (years)		46.0 ± 9.0	52.0 ± 9.0	46.0 ± 9.0	52.0 ± 9.0	98.0 ± 18.0
First Marriage Age (years)		19.0 ± 6.0	18.0 ± 5.0	19.0 ± 6.0	18.0 ± 5.0	37.0 ± 11.0
Physical Activity (MET.h/day)		38.99 ± 6.95	37.92 ± 6.28	38.99 ± 6.94	37.91 ± 6.28	76.91 ± 13.23
Energy intake (Kcal)		3010.85 ± 1027.93	2903.5 0 ± 1085.38	3008.20 ± 1027.43	2906.20 ± 1086.80	5914.35 ± 2113.31
Socio-economic Score		-0.45 ± 1.68	-0.46 ± 1.62	-0.45 ± 1.68	-0.46 ± 1.62	-0.91 ± 3.3
Pregnancies (count)		4.0 ± 3.0	6.0 ± 3.0	4.0 ± 3.0	6.0 ± 3.0	10.0 ± 6.0
Brestfeeding duration (year)		6.29 ± 4.37	8.52 ± 5.34	6.31 ± 4.38	8.50 ± 5.36	14.81 ± 9.71

**Table 2:** Frequency of MetS and its components per number of pregnancies

Number of Pregnancies	MetS (NCEP ATP III criteria)	MetS (IDF criteria)
0	72 (35,1%)	72 (35,1%)
1	51 (29.8%)	51 (29.8%)
2	125 (22.6%)	124 (22.4%)
3	236 (29%)	235 (28.8%)
4	258 (35.4%)	255 (35%)
5	247 (43.5%)	245 (43.1%)
6	223 (48.7%)	223 (48.7%)
7	208 (53.2%)	205 (52.4%)
8	190 (58.1%)	186 (56.9%)
9	161 (63.9%)	160 (63.5%)
10	145 (63.9%)	144 (63.4%)
11	78 (65.5%)	78 (65.5%)
12	144 (72.4%)	142 (71.4%)
OR	1.215	1.212
95%CI-L	1.191	1.188
95%CI-U	1.24	1.236
P_Value	0.000	0.000

Odds ratio (OR) and its’ 95% confidence intervals (95%CI) were computed by univariate logistic regression between MetS as dependent and pregnancy count as continues predictor variables. The frequency of MetS in women with 2 pregnancy was lower than others. Also The odds of MetS increased 1.215 times with each pregnancy

**Table 3:** Frequency of MetS and its components per duration of breast-feeding

Breast feeding duration (Year)	MetS (NCEP ATP III criteria)	MetS (IDF criteria)
0	103 (35.6%)	103 (35.6%)
< 1	50 (36.5%)	50 (36.5%)
1–2	44 (29.5%)	44 (29.5%)
2–3	112 (38.4%)	112 (38.4%)
3–4	89 (28.8%)	88 (28.5%)
4–5	204 (29.8%)	201 (29.3%)
5–6	96 (34.7%)	96 (34.7%)
6–7	228 (36%)	227 (35.9%)
7–8	104 (45.4%)	104 (45.4%)
8–9	177 (42.7%)	176 (42.4%)
9–10	72 (52.9%)	70 (51.5%)
10–11	193 (50.3%)	192 (50%)
> 11	667 (61.8%)	658 (60.9%)
OR	1.121	1.118
95%CI-L	1.103	1.100
95%CI-U	1.138	1.136
P_Value	0.000	0.000

Odds ratio (OR) and its’ 95% confidence intervals (95%CI) were computed by univariate logistic regression between MetS as dependent and breast feeding duration as continues predictor variables. The frequency of MetS in women with 1–2 years breast-feeding was lower than others. Also The odds of MetS increased 1.103 times with each breast-feeding years

**Fig. 1:**
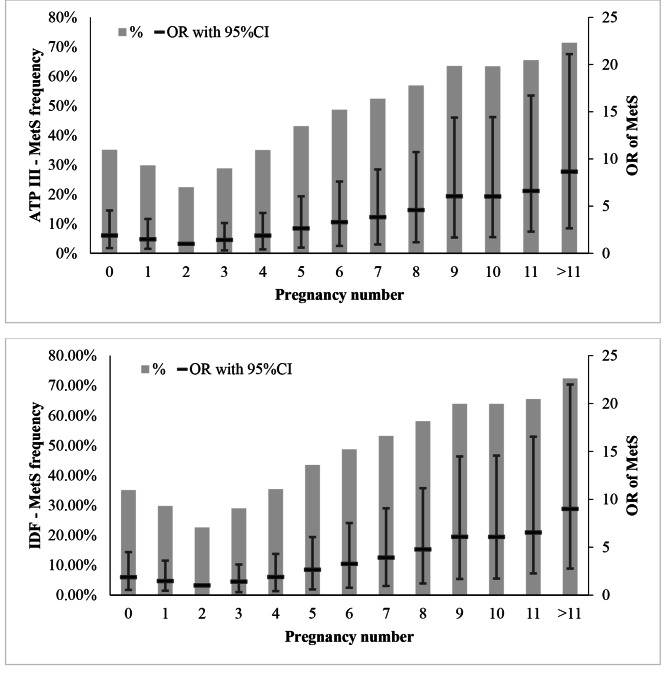
The trend of MetS in women with different pregnancy counts and based on IDF and ATP III methods. The grey bars showed the percentage of MetS and were presented on the left axis. The black lines showed the odds ratio and 95%CI and were presented on the right axis

In Tables 2 and 3, frequency of MetS based on IDF and ATP III methods in different number of pregnancy and breast-feeding years were presented. This presentation were doen for each commponents of MetS in suplementort Tables 1 and 2. Based on the findings presented in Table 2; Fig. 1, the lowest prevalence of MetS was found in women with history of two pregnancies as 22.4% based on IDF criterion, while according to ATP III criterion it was 22.6%. The trend of development of MetS in women with zero to two pregnancies was descending, while after two pregnancies it was ascending. Similar to this phenomenon was also observed for the duration of breast-feeding. Based on ATP III, the lowest prevalence of MetS was observed in groups 1–2 and 4–5, which increases up to 61.8% with prolongation of breast-feeding. According to IDF, a similar trend was observed (Table 4; Fig. 1 -gray bars). Furthermore, logistic regression indicated that with increase in each pregnancy, the chance of developing metabolic syndrome would rise by (OR = 1/118, 95% CI(1/100, 1/136)) based on IDF and by (OR = 1/121, 95% CI(1/103, 1/138)) according to ATP III (Table 2). Similar to this phenomenon was also observed for the duration of breast-feeding (Table 3).

**Table 4:** The results of multivariable logistic regression on the relationship between Pregnancy count MetS adjusted by age, physical activity, energy intake, socio-economic score, marriage age and smoking

	MetS (IDF criteria)				MetS (NCEP ATP III criteria)			
Pregnancy count	OR	95% CI		P_value	OR	95% CI		P_value
		Lower	Upper			Lower	Upper	
0	1.279	0.871	1.876	0.209	1.253	0.854	1.839	0.248
1	1.367	0.914	2.046	0.128	1.350	0.902	2.019	0.145
2 (reference)	1				1			
3	1.319	1.018	1.709	0.036	1.314	1.015	1.701	0.038
4	1.477	1.132	1.927	0.004	1.486	1.140	1.937	0.003
5	1.733	1.305	2.301	< 0.001	1.730	1.303	2.296	< 0.001
6	1.775	1.309	2.406	< 0.001	1.737	1.282	2.354	< 0.001
7	1.643	1.183	2.282	0.003	1.654	1.191	2.296	0.003
8	1.705	1.200	2.422	0.003	1.748	1.230	2.483	0.002
9	2.095	1.421	3.090	< 0.001	2.074	1.406	3.059	< 0.001
10	1.925	1.289	2.876	0.001	1.902	1.273	2.843	0.002
11	1.862	1.137	3.048	0.014	1.800	1.099	2.947	0.019
> 11	2.139	1.367	3.348	0.001	2.176	1.387	3.414	0.001

The history of two pregnancies was chosen as the reference level because of the lower frequency of MetS.

By setting two pregnancies as the criterion and after adjusting for the confounding effect including age, physical activity, energy intake, socioeconomic status, age at first marriage, and cigarette smoking, the chance of developing MetS would diminish non-significantly with increase in the number of pregnancies up to two pregnancies (reference). After this number, with rise in the number of pregnancies, the chance of developing MetS would grow by up to 2.1 times, with all of these increases being significant. This trend was observed in both IDF and ATP III methods (the Figures show pre-adjustment and the table indicates post adjustment). The chance of developing MetS at different breast-feeding intervals after the correction did not have significant differences (Supplementary Tables 4–5).

The frequency of MetS has been shown with bar chart, where the right side axis has been indicated with percentage, the odds ratio has been displayed with horizontal line and its confidence interval has been revealed with vertical line, as indicated in the left axis.

## Discussion and conclusion

This research indicated that 42.3 and 42.7% of 35-70-year-old rural women participating in the study had MetS according to IDF and ATP (III) methods respectively. The chance of developing MetS by setting two pregnancies as the criterion and after adjustment for the confounding effects decreased nonsignificantly with increase in the number of pregnancies up to two. However, beyond this number of pregnancies, the chance of developing MetS grows by up to 2.1 times, with all of these increases being significant. This trend was observed in both MetS calculation methods. The chance of developing MetS in both calculation methods increased within different breast-feeding times for 6–7 years and had some nonsignificant reductions. On the other hand, within the interval of ≥ 7 years, the chance of developing MetS increased significantly to 3.721. Nevertheless eventually, the chance of developing MetS in both calculation methods after the adjustment did not differ significantly.

Previous studies have reported MetS prevalence among women as around 10–60% [20, 38–40]. The reason behind the difference in the reported values of studies could be type of study, selection and volume of samples, examined age groups, racial as well as geographical differences, etc.

The process that occurs during normal pregnancy period can be a temporary transition toward MetS [41]. The factors affecting development of MetS include young age at the time of menstruation, heavy weight, more number of pregnancies, as well as more years of menopause [39]. In spite of 13% increase in the odds of developing MetS per each child, which indicates existence of dose-response relationship, histories of breastfeeding for more than one month would lower this chance and has a protective effect against MetS [20, 38, 40], which is in line with the present study. Also, weight or its variations may be an effective intermediate of breastfeeding in the risk of developing MetS [42].

In this study, prevalence of FBS higher than 100 mg/dl had a descending trend in those up to the second pregnancy, while it increased with further pregnancies.

A cohort study by Naver et al. on Danish women (n = 100,669) with a history of delivery indicated that the risk of diabetes diagnosis would grow in women with increase in the number of pregnancies (more than two pregnancies), which was in line with the present study [43]. Investigation of the large number of subjects above 30 years of age was one of the common points with the present study.

There are numerous studies in line and not in line with these findings [44–46]. The reason of difference in the study results can be type of study, number and selection of samples, examined age groups, racial differences, etc.

In the early stages of pregnancy, insulin sensitivity increases causing absorption of glucose to fat reserves so that the body would become prepared for pregnancy and supply its required energy along pregnancy. With advance of pregnancy and increase of hormones including estrogen, progesterone, leptin, cortisol, the placenta lactogen and placental growth hormone both cause development of an insulin resistance effect, in response to which blood glucose rises slightly, and this glucose is easily transferred through the placenta in order to guarantee the fetal growth. This mild state of insulin resistance also leads to endogenous glucose production and decomposition of fat reserves, thus causing greater elevation of blood glucose and free fatty acids. In most cases, pancreatic cells are not able to compensate the excess fuel chronically, which eventually leads to insulin resistance, hyperglycemia, and increased glucose supply to the growing fetus [46, 47]. At least four pregnancies during reproductive ages may be a potential risk factor for diabetes in menopausal women without a history of gestational diabetes [46].

With increase in the number of pregnancies, the mothers’ weight and eventually the number of diabetics also increase. Meanwhile, it is supposed that breast-feeding can somehow prevent this increase or improve it. This has convinced some researchers to consider pregnancy as a risk factor, and breast-feeding as a protective factor against MetS development [48].

The results of the present study showed that with prolongation of breast-feeding up to 3–4 years, prevalence of FBS diminishes. However, with increase in breast-feeding duration, FBS prevalence grows periodically. Previous studies have also reported findings in line and not in line with the mentioned finding [49–52]. Breast-feeding has been associated with weight loss post-pregnancy and decreased risk of obesity in future, and accordingly it is linked to the main risk factor of diabetes [53]. Complex hormonal changes resulting from pregnancy and effect of breast-feeding on this hormonal environment may influence metabolism of glucose, insulin, and beta cell functions [54–57]. Weight changes postdelivery may mostly be due to hormonal changes / metabolic changes resulting from breast-feeding. Indeed, postdelivery, progesterone secretion stops and the neonate’s nipple suction causes secretion of prolactin. Eventually, the estrogen level drops causing mobility and increased adipose tissue reserves. In addition, since prolactin also inhibits lipogenesis and suppresses glucose absorption in the adipose tissue, it may lead to elevated blood glucose [58]. Furthermore, low levels of estrogen in breast-feeding women may have a protective effect on glucose metabolism and in turn risk of developing diabetes [52].

The results of the present research indicated a downward trend for prevalence of hypertension up to the second pregnancy in the studied individuals. However, with increase in the number of pregnancies, this trend became ascending. Furthermore, breast-feeding for 3–4 years results in diminished prevalence of hypertension and with prolongation of breast-feeding its prevalence grows periodically.

The results of Misty C Day et al. on 34,374 women with a history of pregnancy showed a direct relationship between pregnancy and hypertension, which confirms the present study findings [59]. As a common point with the present research, they also investigated a large number of subjects.

There are numerous studies concurring with and contradicting the results of the present research [60–62].

At the beginning of pregnancy, changes in blood pressure occur as decreased systemic vessel resistance as well as increased stroke volume and cardiac output. Systolic blood pressure remains almost unchanged, but diastolic blood pressure has two states. In the second trimester considering the reduction of the systemic vessel resistance, blood pressure drops by 10 mmHg on average; however, in the third trimester, due to increased blood volume and stroke volume, it returns back to the prepregnancy level [63, 64].

Breast-feeding for more than 12 months was associated with 30% reduction in risk of diabetes, and 13–30% reduction of hypertension in mothers after adjustment for confounding variables. In addition to weight loss, cigarette cessation, and exercise, breast-feeding should also be recommended for mothers because of its advantages [65–67]. Pregnancy and predelivery periods are suitable opportunities for training mothers about the interventions related to lifestyle which are protective for the mothers and child health in the future. In the plan of preventing cardiovascular disease in women, the potential benefits of breast-feeding in cardiovascular health of women should be presented.

The results of some previous studies show that during pregnancy in order to support the fetus and to support for breast-feeding, elevated blood pressure and lipid storage are observed. Breast-feeding within the first weeks to several months postdelivery is associated with decreased blood pressure, and this relationship seems to continue for some time. In case of no breast-feeding, the mother’s body status remains the same, and in case of breast-feeding, it returns back to the prepregnancy state [15, 68, 69]. Furthermore, hormonal mediators including oxytocin hormone in breast-feeding mothers play a key role in reducing systolic and diastolic blood pressure as well as cardiovascular risk factors [61, 70, 71]. Considering the importance and benefits of breast-feeding for the mother’s health in the future, at the time of pregnancy, plans are required regarding duration and extent of breast-feeding and should be trained to mothers [72, 73].

In this research, the minimum HDL level was observed in women with two pregnancies and 3–4 years of breast-feeding. With increase in the number of pregnancies and duration of breast-feeding, HDL was elevated. Numerous studies confirm or reject the findings of this study [74–76]. This difference in the report of study results can be due to the type of study, sample size, age groups, selection of the subjects, racial differences, etc.

In the second and third trimesters of pregnancy, the levels of total cholesterol, LDL, and HDL rise through metabolic events. These changes occur towards accelerating the fetal development as well as energy storage. It may protect both the mother and fetus during long periods of hunger or severe physical activities [77, 78]. The results of previous studies also confirmed the present study findings [79, 80].

High TG prevalence in this study showed a descending trend up to the second pregnancy, while with increase in the number of pregnancies, its prevalence increased. Also, with prolongation of the breast-feeding to 3–4 years, a downward trend was observed in TG prevalence. However, with prolongation of breast-feeding, its prevalence increased periodically. The results of some previous studies concur and some others disagree with the present research [20, 76].

Hyperlipidemia in the second half of pregnancy is considered a physiological mechanism required for maintaining fuel supplement for the fetus. Nevertheless, it may be a pathological finding which indicates development of MetS. Previous studies have indicated that the blood lipid level declines at the beginning of pregnancy, after which a gradual rise occurs, and at the end of the third trimester it peaks, while the lipid levels drop within four months postdelivery [81].

During pregnancy, the hormonal changes that occur cause insulin resistance and reduced activity of lipoprotein lipase (LPL), and ultimately 2-3-fold increase in the mother’s triglyceride level [74, 82–84]. Also, changes of TG value are directly affected by the person’s nutritional levels [85]. The fat reserves develop during pregnancy considering prediction of the mother’s metabolic demand at the time of breastfeeding. Now, if breastfeeding does not occur, these metabolic changes continue, causing adverse health consequences for the mother [86]. The results of animal studies have also reported this causal relationship between breastfeeding and fat reserves [87].

In the studied individuals, the waist circumference showed a descending trend until the second pregnancy, while with increase of pregnancies, its value increased. Further, with prolongation of breast-feeding to 3–4 years, the waist circumference revealed a descending trend; however, with prolongation of breast-feeding its value would grow periodically [65, 88, 89].

Over recent decades, the number of women with overweight and obesity during reproductive ages has increased considerably. These individuals are more susceptible to reduced insulin sensitivity, and hence increased risk of developing metabolic disorders (including gestational diabetes, preeclampsia, and fetal overgrowth) [90]. It is suggested that pregnancies be planned and weight control should also be done before pregnancy, in order to prevent excessive weight gain during pregnancy, post-delivery, along with its complications.

### Strengths and limitations

The strong points of this study included the large sample size (n = 5509) as well as extensive measurement of confounding factors in the Fasa Persian cohort study, which contributed to precise examination of the relationship between number of pregnancies and MetS. One limitation of this research was its cross-sectional nature which would not allow us to draw definite conclusions or causal relationships. It is suggested to employ longitudinal and prospective models for better assessment of results.

No information was available about the time of the last delivery. Regarding duration of breastfeeding, subjects’ recall may have been prone to errors, since it was memory based.

## Conclusion

Increase in the number of pregnancies to more than twice and breast-feeding of more than seven years would elevate the chance of developing MetS based on both IDF and ATP III methods. Thus, it can probably be concluded that the copresence of metabolic syndrome and its elements in pregnancy has consequences for both the mother and fetus. Thus, it is recommended that under pregnancy conditions by investigating the status of metabolic syndrome and its components in mothers as well as in different parts of the country, solutions can be presented for optimizing and preventing untoward consequences. The results of epidemiological studies and animal models indicated that breast-feeding may be modifiable as a preventive factor for risk of MetS. If this causal relationship is considered, in future planning and policymaking, it is suggested that women with pregnancies of more than twice or long duration of breast-feeding undergo specialized examinations and monitoring to investigate and control MetS problems in order to prevent future problems.

## Electronic supplementary material

Below is the link to the electronic supplementary material.

**Additional File 1:** Detailed analyzes regarding relationship the number of pregnancies and components of the metabolic syndrome

## Data Availability

The datasets used and/or analyzed during the current study are available from the corresponding author on request.
