# Supplementary material for: The relationship between pregnancy count and duration of breast-feeding with metabolic syndrome (Fasa Persian cohort study)
Source: BMC Womens Health. 2023 Jul 13;23:371. doi: 10.1186/s12905-023-02528-4 (PMC10339557; doi:10.1186/s12905-023-02528-4)
Supplement: Supplementary file 1 — Additional File 1: Detailed analyzes regarding relationship the number of pregnancies and components of the metabolic syndrome [file 12905_2023_2528_MOESM1_ESM.docx]

Supplementary Table 1. Frequency of each MetS components per numbers of pregnancy

|  | | **Diabetes**  **N=1212** | **HTN**  **N= 2021** | **TG**  **N= 1388** | **HDL**  **N= 2553** | **Waist Circumference**  **N= 4701** | **The affected component count of MetS** | | | | | |
| --- | --- | --- | --- | --- | --- | --- | --- | --- | --- | --- | --- | --- |
|  |  |  |  |  |  |  | **0**  **N= 81** | **1**  **N=960** | **2**  **N=1835** | **3**  **N=1417** | **4**  **N=611** | **5**  **N=110** |
| Number of Pregnancies | 0 | 37  (18%) | 68  (33,2%) | 60  (29,3%) | 119  (58%) | 177  (86,3%) | 5  (2,4%) | 41  (21%) | 87  (42,4%) | 46  )22,4%) | 22  )10,7%) | 4  (2%) |
|  | 1 | 21  (12.3%) | 45  (26.3%) | 43  (25.1%) | 80  (46.8%) | 154  (90.1%) | 7  (4.1%) | 52  (30.4%) | 61  (35.7%) | 37  (21.6%) | 12  (7%) | 2  (1.2%) |
|  | 2 | 74  (13.4%) | 115  (20.8%) | 119  (21.5%) | 256  (46.2%) | 520  (93.9%) | 10  (1.8%) | 167  (30.1%) | 252  (45.5%) | 92  (16.6%) | 28  (5.1%) | 5  (0.9%) |
|  | 3 | 125  (15.3%) | 206  (25.3%) | 170  (20.9%) | 407  (49.9%) | 766  (94%) | 16  (2%) | 217  (26.6%) | 346  (42.5%) | 183  (22.5%) | 49  (6%) | 4  (0.5%) |
|  | 4 | 138  (19%) | 228  (31.3%) | 183  (25.1%) | 358  (49.2%) | 679  (93.3%) | 14  (1.9%) | 175  (24%) | 281  (38.6%) | 195  (26.8%) | 51  (7%) | 12  (1.6%) |
|  | 5 | 129  (22.7%) | 224  (39.4%) | 164  (28.9%) | 287  (50.5%) | 536  (94.4%) | 12  (2.1%) | 101  (17.8%) | 208  (36.6%) | 172  (30.3%) | 68  (12%) | 7  (1.2%) |
|  | 6 | 122  (26.6%) | 214  (46.7%) | 136  (29.7%) | 245  (53.5%) | 433  (94.5%) | 6  (1.3%) | 67  (14.6%) | 162  (35.4%) | 147  (32.1%) | 62  (13.5%) | 14  (3.1%) |
|  | 7 | 127  (32.5%) | 202  (51.7%) | 115  (29.4%) | 223  (57%) | 371  (94.9%) | 0  (0%) | 39  (10%) | 144  (36.8%) | 132  (33.8%) | 65  (16.6%) | 11  (2.8%) |
|  | 8 | 121  (37%) | 178  (54.4%) | 115  (35.2%) | 174  (53.2%) | 302  (92.4%) | 5  (1.5%) | 37  (11.3%) | 95  (29.1%) | 113  (34.6%) | 61  (18.7%) | 16  (4.9%) |
|  | 9 | 86  (34.1%) | 167  (66.3%) | 96  (38.1%) | 128  (50.8%) | 239  (94.8%) | 2  (0.8%) | 23  (9.1%) | 66  (26.2%) | 92  (36.5%) | 60  (23.8%) | 9  (3.6%) |
|  | 10 | 102  (44.9%) | 152  (67%) | 71  (31.3%) | 113  (49.8%) | 214  (94.3%) | 2  (0.9%) | 18  (7.9%) | 62  (27.3%) | 80  (35.2%) | 55  (24.2%) | 10  (4.4%) |
|  | 11 | 43  (36.1%) | 80  (67.2%) | 43  (36.1%) | 65  (54.6%) | 118  (99.2%) | 0  (0%) | 10  (8.4%) | 31  (26.1%) | 42  (35.3%) | 29  (24.4%) | 7  (5.9%) |
|  | 12 | 87  (43.7%) | 142  (71.4%) | 73  (36.7%) | 98  (49.2%) | 192  (96.5%) | 2  (1%) | 13  (6.5%) | 40  (20.1%) | 86  (43.2%) | 49  (24.6%) | 9  (4.5%) |
|  | OR | 1.179 | 1.242 | 1.074 | 1.012 | 1.079 |  | | | | | |
|  | 95%CI-L | 1.154 | 1.217 | 1.053 | 0.993 | 1.037 |  |  |  |  |  |  |
|  | 95%CI-U | 1.205 | 1.268 | 1.096 | 1.031 | 1.124 |  |  |  |  |  |  |
|  | P_Value | 0.000 | 0.000 | 0.000 | 0.211 | 0.000 |  | | | | | |

Odds ratio (OR) and its’ 95% confidence intervals (95%CI) were computed by univariate logistic regression between each MetS components as dependent and pregnancy count as continues predictor variables.

Supplementary Table 2. Frequency of each MetS components per duration of breast-feeding

|  | | **Diabetes**  **N=1212** | **HTN**  **N= 2022** | **TG**  **N= 1388** | **HDL**  **N= 2554** | **Waist Circumference**  **N= 4702** | **The affected component count of MetS** | | | | | |  |
| --- | --- | --- | --- | --- | --- | --- | --- | --- | --- | --- | --- | --- | --- |
|  |  |  |  |  |  |  | **0**  **N= 81** | **1**  **N=960** | **2**  **N=1835** | **3**  **N=1418** | **4**  **N=611** | **5**  **N=110** | |
| Breast feeding duration (Year) | 0 | 53  (18.3 %) | 95  (32.9 %) | 89  (30.8 %) | 157  (54.3 %) | 260  (90 %) | 6  (2.1%) | 55  (19 %) | 125  (43.3 %) | 69  (23.9 %) | 28  (9.7 %) | 6  (2.1%) | |
|  | <1 | 30  (21.9 %) | 46  (33.6 %) | 31  (22.6 %) | 75  (54.7 %) | 127  (92.7 %) | 5  (3.6%) | 25  (18.2 %) | 57  (41.6%) | 32  (23.4 %) | 16  (11.7 %) | 2  (1.5%) | |
|  | 1-2 | 32  (21.5 %) | 40  (26.8 %) | 32  (21.5 %) | 69  (46.3 %) | 128  (85.9 %) | 10  (6.7%) | 43  (28.9 %) | 52  (34.9 %) | 25  (16.8 %) | 16  (10.7 %) | 3  (2 %) | |
|  | 2-3 | 67  (22.9 %) | 94  (32.2 %) | 76  (26 %) | 147  (50.3 %) | 272  (93.2 %) | 4  (1.4%) | 76  (26 %) | 100  (34.2 %) | 76  (26 %) | 28  (9.6 %) | 8  (2.7%) | |
|  | 3-4 | 46  (14.9 %) | 87  (28.2 %) | 60  (19.4 %) | 138  (44.7 %) | 292  (94.5 %) | 6  (1.9%) | 99  (32 %) | 115  (37.2 %) | 63  (20.4 %) | 25  (8.1 %) | 1  (0.3%) | |
|  | 4-5 | 113  (16.5 %) | 194  (28.3 %) | 153  (22.3 %) | 339  (49.5 %) | 646  (94.3 %) | 8  (1.2%) | 171  (25 %) | 302  (44.1 %) | 153  (22.3 %) | 44  (6.4 %) | 7  (1%) | |
|  | 5-6 | 44  (15.9 %) | 82  (29.6 %) | 76  (27.4 %) | 147  (53.1 %) | 262  (94.6 %) | 4  (1.4%) | 69  (24.9 %) | 108  (39 %) | 65  (23.5 %) | 24 (8.7 %) | 7  (2.5%) | |
|  | 6-7 | 119  (18.8 %) | 197  (31.1 %) | 180  (28.4 %) | 306  (48.3 %) | 594  (93.8 %) | 14  (2.2%) | 137  (21.6 %) | 254  (40.1 %) | 170  (26.9 %) | 49  (7.7 %) | 9  (1.4%) | |
|  | 7-8 | 59  (25.8 %) | 97  (42.4 %) | 59  (25.8 %) | 123  (53.7 %) | 220  (96.1 %) | 3  (1.3%) | 34  (14.8 %) | 88  (38.4 %) | 71  (31 %) | 30  (13.1 %) | 3  (1.3%) | |
|  | 8-9 | 95  (22.9 %) | 154  (37.1 %) | 112  (27 %) | 212  (51.1 %) | 387  (93.3 %) | 7  (1.7%) | 82  (19.8 %) | 149  (35.9 %) | 134  (32.3 %) | 37  (8.9 %) | 6  (1.4%) | |
|  | 9-10 | 44  (32.4 %) | 73  (53.7 %) | 41  (30.1 %) | 69  (50.7 %) | 130  (95.6 %) | 1  (0.7%) | 17  (12.5 %) | 46  (33.8 %) | 47  (34.6 %) | 18  (13.2 %) | 7  (5.1%) | |
|  | 10-11 | 107  (27.9 %) | 183  (47.7 %) | 123  (32 %) | 207  (53.9 %) | 364  (94.8 %) | 7  (1.8%) | 39  (10.2 %) | 145  (37.8 %) | 126  (32.8 %) | 58  (15.1 %) | 9  (2.3%) | |
|  | >11 | 403  (37.3 %) | 680  (63 %) | 356  (33 %) | 565  (52.3 %) | 1020  (94.4 %) | 6  (0.6%) | 113  (10.5 %) | 294  (27.2 %) | 387  (35.8 %) | 238  (22 %) | 42(3.9%) | |
|  | OR | 1.101 | 1.139 | 1.041 | 1.008 | 1.049 |  | | | | | |  |
|  | 95%CI-L | 1.081 | 1.121 | 1.032 | 0.993 | 1.017 |  |  |  |  |  |  |  |
|  | 95%CI-U | 1.122 | 1.158 | 1.058 | 1.023 | 1.081 |  |  |  |  |  |  |  |
|  | P_Value | 0.000 | 0.000 | 0.00 | 0.286 | 0.002 |  |  |  |  |  |  |  |

Odds ratio (OR) and its’ 95% confidence intervals (95%CI) were computed by univariate logistic regression between each MetS components as dependent and duration of breast-feeding years as continues predictor variables.

Supplementary Table 3 - The results of logistic regression on the relationship between pregnancy count and MetS (before adjustment)

|  | MetS (IDF criteria) | | | | MetS (NCEP ATP III criteria) | | | | |
| --- | --- | --- | --- | --- | --- | --- | --- | --- | --- |
| Pregnancy Number | OR | 95 % OR | | P_value | OR | 95 % OR | | P_value |  |
|  |  | Lower | Upper |  |  | Lower | Upper |  |  |
| 0 | 1.877 | 1.324 | 2.663 | <0.001 | 1.858 | 1.310 | 2.634 | 0.001 |  |
| 1 | 1.474 | 1.004 | 2.163 | 0.048 | 1.459 | 0.994 | 2.140 | 0.054 |  |
| Reference (2) | 1 |  |  |  | 1 |  |  |  |  |
| 3 | 1.405 | 1.093 | 1.805 | 0.008 | 1.399 | 1.089 | 1.797 | 0.009 |  |
| 4 | 1.870 | 1.454 | 2.403 | <0.001 | 1.884 | 1.466 | 2.420 | <0.001 |  |
| 5 | 2.630 | 2.029 | 3.411 | <0.001 | 2.641 | 2.038 | 3.422 | <0.001 |  |
| 6 | 3.291 | 2.509 | 4.315 | <0.001 | 3.257 | 2.484 | 4.269 | <0.001 |  |
| 7 | 3.822 | 2.884 | 5.065 | <0.001 | 3.901 | 2.944 | 5.168 | <0.001 |  |
| 8 | 4.574 | 3.401 | 6.152 | <0.001 | 4.760 | 3.538 | 6.403 | <0.001 |  |
| 9 | 6.031 | 4.357 | 8.348 | <0.001 | 6.072 | 4.386 | 8.406 | <0.001 |  |
| 10 | 6.016 | 4.300 | 8.419 | <0.001 | 6.069 | 4.336 | 8.494 | <0.001 |  |
| 11 | 6.597 | 4.302 | 10.117 | <0.001 | 6.529 | 4.259 | 10.010 | <0.001 |  |
| >11 | 8.639 | 5.988 | 12.464 | <0.001 | 8.986 | 6.212 | 12.997 | <0.001 |  |

The history of two pregnancies was chosen as the reference level because of the lower frequency of MetS.

Supplementary Table 4 - The results of logistic regression for the relationship between duration of breast-feeding and MetS (before adjustment)

|  | MetS (IDF criteria) | | | | MetS (NCEP ATP III criteria) | | | | |
| --- | --- | --- | --- | --- | --- | --- | --- | --- | --- |
| Breast feeding duration (year) | OR | 95 % CI | | P_value | OR | 95 % CI | | P_value |  |
|  |  | Lower | Upper |  |  | Lower | Upper |  |  |
| 0 | 1.321 | 0.863 | 2.024 | 0.200 | 1.321 | 0.863 | 2.024 | 0.200 |  |
| <1 | 1.371 | 0.836 | 2.250 | 0.211 | 1.371 | 0.836 | 2.250 | 0.211 |  |
| Reference (1-2) | 1 |  |  |  | 1 |  |  |  |  |
| 2-3 | 1.485 | 0.972 | 2.268 | 0.067 | 1.485 | 0.972 | 2.268 | 0.067 |  |
| 3-4 | 0.950 | 0.618 | 1.461 | 0.816 | 0.965 | 0.628 | 1.483 | 0.872 |  |
| 4-5 | 0.991 | 0.672 | 1.462 | 0.964 | 1.012 | 0.686 | 1.492 | 0.952 |  |
| 5-6 | 1.266 | 0.823 | 1.946 | 0.283 | 1.266 | 0.823 | 1.946 | 0.283 |  |
| 6-7 | 1.334 | 0.905 | 1.966 | 0.145 | 1.343 | 0.912 | 1.979 | 0.135 |  |
| 7-8 | 1.985 | 1.282 | 3.076 | 0.002 | 1.985 | 1.282 | 3.076 | 0.002 |  |
| 8-9 | 1.757 | 1.175 | 2.627 | 0.006 | 1.775 | 1.187 | 2.653 | 0.005 |  |
| 9-10 | 2.531 | 1.556 | 4.118 | <0.001 | 2.685 | 1.649 | 4.370 | <0.001 |  |
| 10-11 | 2.386 | 1.592 | 3.577 | <0.001 | 2.411 | 1.609 | 3.615 | <0.001 |  |
| >11 | 3.721 | 2.563 | 5.401 | <0.001 | 3.854 | 2.655 | 5.595 | <0.001 |  |

The 1-2 years breast-feeding was chosen as the reference level because of the lower frequency of MetS.

Supplementary Table 5 - The results of multivariate logistic regression for the relationship between duration of breast-feeding and MetS adjusted by age, physical activity, energy intake, socio-economic score, marriage age and smoking

|  | MetS (IDF criteria) | | | | MetS (NCEP ATP III criteria) | | | | |
| --- | --- | --- | --- | --- | --- | --- | --- | --- | --- |
| Breast feeding duration (year) | OR | 95 % CI | | P_value | OR | 95 % CI | | P_value |  |
|  |  | Lower | Upper |  |  | Lower | Upper |  |  |
| 0 | 1.329 | 0.841 | 2.102 | 0.223 | 1.326 | 0.838 | 2.098 | 0.227 |  |
| <1 | 1.443 | 0.856 | 2.432 | 0.169 | 1.442 | 0.855 | 2.431 | 0.170 |  |
| 1-2 (reference) | 1 |  |  |  | 1 |  |  |  |  |
| 2-3 | 1.598 | 1.024 | 2.493 | 0.039 | 1.603 | 1.027 | 2.501 | 0.038 |  |
| 3-4 | 1.044 | 0.665 | 1.640 | 0.851 | 1.066 | 0.679 | 1.674 | 0.782 |  |
| 4-5 | 1.215 | 0.809 | 1.825 | 0.349 | 1.247 | 0.830 | 1.874 | 0.288 |  |
| 5-6 | 1.208 | 0.768 | 1.900 | 0.413 | 1.212 | 0.771 | 1.907 | 0.404 |  |
| 6-7 | 1.388 | 0.924 | 2.085 | 0.115 | 1.404 | 0.934 | 2.109 | 0.103 |  |
| 7-8 | 1.440 | 0.907 | 2.284 | 0.122 | 1.440 | 0.907 | 2.285 | 0.122 |  |
| 8-9 | 1.326 | 0.867 | 2.027 | 0.193 | 1.340 | 0.876 | 2.049 | 0.177 |  |
| 9-10 | 1.303 | 0.779 | 2.181 | 0.314 | 1.382 | 0.825 | 2.314 | 0.219 |  |
| 10-11 | 1.298 | 0.842 | 1.999 | 0.237 | 1.307 | 0.848 | 2.014 | 0.225 |  |
| >11 | 1.418 | 0.942 | 2.136 | 0.095 | 1.462 | 0.970 | 2.202 | 0.069 |  |

The 1-2 years breast-feeding was chosen as the reference level because of the lower frequency of MetS.
